# Spontaneous Rupture of the Renal Pelvis Due to Extrinsic Obstruction by Metastatic Retroperitoneal Lymphadenopathy

**DOI:** 10.7759/cureus.22986

**Published:** 2022-03-09

**Authors:** Benjamin A Fink, Young Son, Ronald Clearie, Bum Kim, Thomas J Mueller

**Affiliations:** 1 Urology, Rowan University School of Osteopathic Medicine, Stratford, USA; 2 Urology, Jefferson Stratford Hospital, Stratford, USA; 3 Urology, New Jersey Urology, Voorhees, USA

**Keywords:** ureteric obstruction, forniceal rupture, spontaneous rupture, renal pelvis, retroperitoneal lymphadenopathy

## Abstract

Spontaneous rupture of the renal pelvis due to metastatic disease is a rare complication. Renal pelvis rupture often goes undiagnosed in cases of non-traumatic origin due to its vague abdominal and flank symptoms. We present a case of an 81-year-old male with primary non-small cell lung cancer who had renal pelvis rupture due to extrinsic compression of the ureter by retroperitoneal lymphadenopathy secondary to metastatic disease.

## Introduction

Spontaneous rupture of the renal pelvis without forniceal rupture is a rare event and has been reported with urolithiasis [1]. In the setting of obstruction, contrast extravasation is often due to forniceal rupture from elevated intrarenal pressures in the collecting system and can be associated with malignant compression, bladder outlet obstruction, and ureteropelvic junction obstruction [2]. The ureter is composed of three layers, including the inner mucosa, muscle layer, and outer serosa [3]. The distal ureter contains three smooth muscle layers, including the inner longitudinal, middle circular, and outer longitudinal layers [4]. The proximal ureter, including the renal pelvis, is composed of only two smooth muscle layers, consisting of the inner longitudinal and outer circular muscle layers [4]. The two-layered proximal ureter is susceptible to damage as studies have found there to be decreased circumferential tensile strength in proximal portions of the ureter [5].

Spontaneous rupture of the pelvicalyceal system is most often precipitated by evidence of hydroureteronephrosis [6]. Hydroureteronephrosis, which can be found incidentally on cross-sectional imaging, often resolves with treatment of the underlying cause; however, evidence of hydroureteronephrosis with concomitant renal pelvis rupture has also been described in literature [7]. Diagnosis of renal pelvis rupture is performed on computerized tomography (CT) with delayed phase with contrast extravasation seen in the retroperitoneal, perinephric, or peripelvic spaces [8]. 

We present a unique case of unilateral renal pelvic rupture secondary to extrinsic obstruction from retroperitoneal lymphadenopathy of primary lung metastases.

## Case presentation

This patient is an 81-year-old male with a past medical history of hypertension, duodenal arteriovenous malformations (AVMs), and hyperlipidemia who presented to the emergency department (ED) with a chief complaint of generalized weakness and difficulty swallowing for the past few weeks. On presentation, the patient's creatinine was 1.55 mg/dL, white blood cell (WBC) count of 27.7 x 10^3^ per uL, hemoglobin of 8.6 g/dL, and a lactate of 2.1 mg/dL. Initial blood and urine cultures performed in the ED were negative. The patient was started on empiric piperacillin-tazobactam due to his leukocytosis and lactate levels. Cervical and supraclavicular adenopathy was noted on the physical exam. A CT of the soft tissue of the neck without contrast was performed, revealing a soft tissue mass in the left supraclavicular region, potentially representing enlarged lymphadenopathy. 

**Figure 1:**
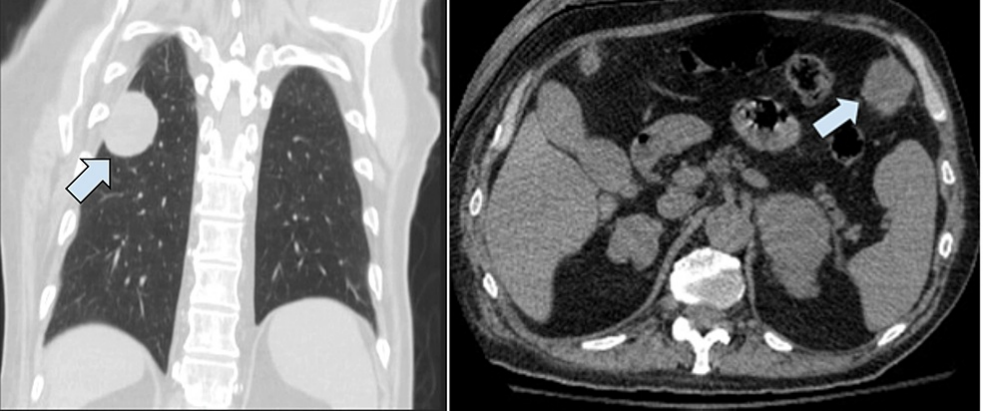
Cross-sectional CT imaging CT cross-sectional imaging in the coronal plane (left panel) of a well-circumscribed peripheral nodularity measuring 4.3 x 3.3 x 4 cm (arrow). Cross-sectional CT imaging in transverse section (right panel) of an enlarged nodularity in left anterior upper abdomen measuring 3.5 x 3.1 cm (arrow).

Three weeks prior, the patient had plain radiograph imaging for a fall. A right humeral head fracture was discovered as well as an incidental finding of a well-defined mass in the right midlung field measuring 5 x 4.6 cm. Further imaging via CT without contrast revealed multiple soft tissue nodules in the chest wall and upper abdomen, concerning for metastatic disease (Figure 1).

The patient was admitted for leukocytosis, anemia, acute kidney injury, and dysphagia. On admission, the empiric antibiotics (piperacillin-tazobactam) started in the ED were discontinued despite the leukocytosis due to the patient's history of polyclonal gammopathy and history of leukocytosis from prior hospitalizations. On the third day in the hospital, the patient's WBC count increased to 28.1 x 10^3^ per uL, with a serum lactate of 4.5 mg/dL in association with tachypnea and hypotension. Repeat blood and urine cultures were performed due to suspicion of infection, and the patient was upgraded to the intensive care unit. The patient was started on vancomycin and restarted on piperacillin-tazobactam empirically. Creatinine was noted to be elevated at 2.42 mg/dL, as well as a hemoglobin count of 6.8 g/dL, necessitating transfusion of one unit of red blood cells. The acute kidney injury was suspected to be prerenal in nature due to intravascular depletion and poor oral intake. Intravenous (IV) fluid resuscitation was implemented.

On the fourth day in the hospital, the patient's WBC count increased to 70 x 10^3^ per uL, lactate dehydrogenase (LDH) of 630 U/L, creatinine of 3.05 mg/dL, and a lactate of 2.2 mg/dL. Repeat blood cultures taken one day prior were positive for *Klebsiella pneumoniae*. Gentamicin was added to the antibiotic regimen based on culture sensitivities. After stabilization, interventional radiology fine-needle core biopsy was performed on the left supraclavicular lesion. Cytology of the biopsy revealed poorly-differentiated adenocarcinoma positive for malignancy.

**Figure 2:**
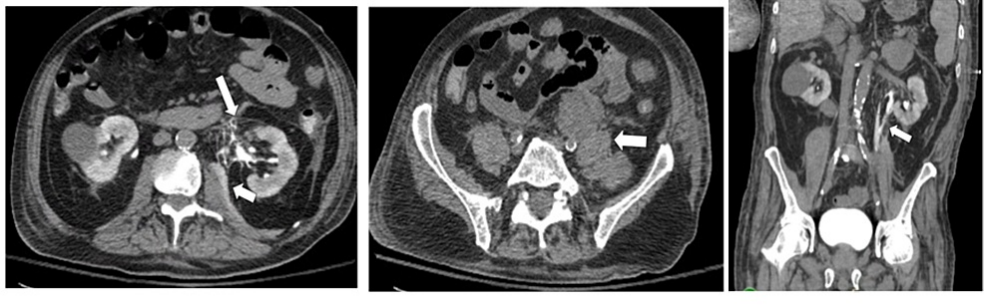
CT scan of the abdomen and pelvis CT abdomen and pelvis (CTAP) in transverse plane (left panel) showing extravasation of contrast at the anteromedial aspect of the left renal pelvis (long arrow) with extension along the left iliopsoas muscle (short arrow). Transverse plane CTAP without contrast (middle panel) displaying compression of the left distal ureter by enlarged retroperitoneal lymph nodes measuring 10 x 4 cm (arrow). Coronal plane view of CTAP without contrast (right panel) showing extravasation of retained contrast medial to the renal pelvis outlining the iliopsoas (arrow).

The patient's WBC count, creatinine, and lactate levels began to downtrend on the fifth day in the hospital, with a WBC count of 50.2 x 10^3^ per uL, creatinine of 2.81 mg/dL, and a lactate of 1.4 mg/dL. However, the patient reported new-onset episodes of non-bilious, non-bloody vomiting as well as worsening right-sided abdominal pain rated as a 9.5 of 10. A CT of the abdomen and pelvis without contrast revealed extravasation of contrast at the level of the renal pelvis with the contrast noted along the left iliopsoas muscle and the left anterior pararenal space suggestive of renal pelvis rupture. Contrast was also identified medially, adjacent to the renal pelvis along the perirenal space, supporting the diagnosis of renal pelvis rupture. Although a non-contrast CT was performed, retained contrast was present from a CT angiography (CTA) with intravenous (IV) contrast conducted two days prior. The IV contrast had delayed excretion from the left renal collecting system due to presumed obstruction. Compression of the left distal ureter was caused by enlarged retroperitoneal lymphadenopathy. Additionally, a large conglomerate of enlarged lymph nodes was found along the left iliopsoas muscle measuring 10 x 4 cm (Figure 2). 

After a discussion with the patient's family, the patient was ultimately placed on comfort care measures, and further surgical intervention was not pursued. The patient expired on the sixth day of hospital stay, one day after the CT scan showing extravasation of contrast of the renal pelvis.

## Discussion

Malignant extrinsic compression of the ureter has been documented as a cause of renal forniceal rupture but has never been reported to cause renal pelvis rupture [2]. In renal forniceal rupture, imaging would reveal primary contrast extravasation along the perirenal space with possible tracking down the psoas; however, in this case, primary contrast extravasation was not found in the perirenal space to support this diagnosis [9]. We suspect that chronic extrinsic compression of the distal ureter via retroperitoneal lymphadenopathy resulted in long-standing obstructive uropathy, ultimately causing a spontaneous pelvic rupture. A proposed mechanism of renal pelvis rupture is obstruction precipitating dilation and increasing the intraluminal pressure that exceeds transitional epithelial and connective tissue integrity [1]. 

In chronic hydronephrosis, the renal pelvis can become thinner, ischemic, and susceptible to damage [10]. There are several sequelae after urinary extravasation, including nonspecific abdominal pain, flank pain, nausea, and vomiting [7]. Furthermore, electrolyte abnormalities can occur due to the reabsorption of urine in the setting of extravasation [11]. We believe the elevated creatinine level in our patient was due to the reabsorption of urine and creatinine from the malignant compression of the ureter and urinary extravasation [11,12]. 

CT urogram is the most sensitive diagnostic modality for renal collecting system rupture [11]. Classically, a renal pelvis rupture would reveal perinephric stranding and contrast extravasation along the perinephric, peripelvic, or retroperitoneal spaces [8]. Although a non-contrast CT was performed in this patient, imaging showed contrast extravasation from the retained IV contrast used in a previous CTA. This retained contrast provided a serendipitous delayed phase of CT abdomen and pelvis. CT imaging revealed contrast extravasation along the left anterior pararenal space with coursing along the ipsilateral iliopsoas, highly suggestive of a renal pelvis rupture. 

Conservative treatment for spontaneous rupture of the renal pelvis in the acute setting includes urinary diversion with a nephroureteral stent and broad-spectrum antibiotics [11]. If a nephroureteral stent cannot be placed, a percutaneous nephrostomy tube is recommended [11]. In this case, there was no treatment, so we were unable to assess the effectiveness of a nephroureteral stent or percutaneous nephrostomy tube. If there is significant urinary extravasation noted, interventional radiology could place a drain. In the event of emergent collecting duct disruption, surgical intervention is required, especially in the setting of trauma [8]. Renal artery embolization, renal repair, and nephrectomy have all been cited as treatment alternatives in the literature if conservative treatment methods fail [6,13]. In the setting of metastatic non-small cell lung cancer, the aggressive treatment options were unwanted, and comfort measures were taken in our patient. 

Metastasis to the retroperitoneum in non-small lung cancer is rare but has been reported [14]. The most common locations for non-small cell lung cancer metastasis include bone, brain, liver, and adrenal glands [15]. Metastatic ureteral involvement of primary non-small cell lung also has been discussed in the literature, but is not predicted to be the source of the renal pelvis rupture in this case. Kodama et al. described a patient with known non-small cell lung cancer presenting with a retroperitoneal mass via a scirrhous spread pattern and hydronephrosis [16]. In our patient, we believe the retroperitoneal lymphadenopathy was secondary to metastatic spread of diagnosed primary non-small cell lung cancer. However, it is unknown if the patient’s metastatic non-small cell lung cancer locally invaded the collecting system making it susceptible for rupture.

## Conclusions

Spontaneous rupture of the renal pelvis is a rare complication seldom discussed in the literature. Urinary tract obstruction due to urolithiasis is an identified risk factor of this phenomenon. We present a unique cause of collecting system violation via retroperitoneal lymphadenopathy secondary to suspected lung metastasis. The treatment of urinary collecting system rupture can be managed with a retrograde nephroureteral stent if access is achieved. A percutaneous nephrostomy tube is the next line of treatment if a retrograde nephroureteral stent is not feasible or in the case of complete avulsion. Open surgical interventions can be utilized in unstable urinary collecting system rupture or if conservative treatment methods fail. We recommend increasing surveillance via radiographic imaging in patients with known metastatic lymphadenopathy to hasten detection for appropriate intervention.
